# Biomimetic Ceramic Composite: Characterization, Cell Response, and In Vivo Biocompatibility

**DOI:** 10.3390/ma14237374

**Published:** 2021-12-01

**Authors:** Hung-Yang Lin, Yi-Jung Lu, Hsin-Hua Chou, Keng-Liang Ou, Bai-Hung Huang, Wen-Chien Lan, Takashi Saito, Yung-Chieh Cho, Yu-Hsin Ou, Tzu-Sen Yang, Pei-Wen Peng

**Affiliations:** 1 Department of Dentistry, Fu Jen Catholic University Hospital, Fu Jen Catholic University, New Taipei City 242, Taiwan; a00207@mail.fjuh.fju.edu.tw; 2 Division of Family and Operative Dentistry, Department of Dentistry, Taipei Medical University Hospital, Taipei 110, Taiwan; yi_jung2002@yahoo.com.tw; 3 School of Oral Hygiene, College of Oral Medicine, Taipei Medical University, Taipei 110, Taiwan; hhchou@tmu.edu.tw; 4 Dental Department of Wan-Fang Hospital, Taipei Medical University, Taipei 116, Taiwan; 5 Biomedical Technology R & D Center, China Medical University Hospital, Taichung 404, Taiwan; klou@tmu.edu.tw (K.-L.O.); u109312001@cmu.edu.tw (B.-H.H.); D204106003@tmu.edu.tw (Y.-C.C.); 6 Department of Oral Hygiene Care, Ching Kuo Institute of Management and Health, Keelung 203, Taiwan; jameslan@ems.cku.edu.tw; 7 Department of Dentistry, Taipei Medical University-Shuang Ho Hospital, New Taipei City 235, Taiwan; 8 Division of Clinical Cariology and Endodontology, Department of Oral Rehabilitation, School of Dentistry, Health Sciences University of Hokkaido, Hokkaido 061-0293, Japan; t-saito@hoku-iryo-u.ac.jp; 9 3D Global Biotech Inc., New Taipei City 221, Taiwan; 10 Taiwan Society of Blood Biomaterials, New Taipei City 221, Taiwan; 11 Graduate Institute of Dental Science, College of Dentistry, China Medical University, Taichung 404, Taiwan; 12 School of Dentistry, College of Oral Medicine, Taipei Medical University, Taipei 110, Taiwan; 13 Excelsior School, Arcadia, CA 91007, USA; jennifer30526@gmail.com; 14 Research Center for Biomedical Devices and Prototyping Production, Taipei Medical University, Taipei 110, Taiwan; 15 Graduate Institute of Biomedical Optomechatronics, College of Biomedical Engineering, Taipei Medical University, Taipei 110, Taiwan; 16 School of Dental Technology, College of Oral Medicine, Taipei Medical University, Taipei 110, Taiwan

**Keywords:** bioactive materials, bioceramics, biocompatibility, composites, calcium phosphate

## Abstract

The present study aimed to synthesize biphasic calcium phosphate ceramics (CaPs) composed of β-tricalcium phosphate (β-TCP) and hydroxyapatite (HAp) from the propagated *Scleractinian* coral and dicalcium phosphate anhydrous using a solid-state reaction followed by heat treatment at a temperature of 1100 °C for 1 h to 7 days. The as-prepared coral and coral-derived biphasic CaPs samples were characterized through scanning electron microscopy, X-ray diffractometry, Fourier transform infrared spectroscopy, and Raman spectroscopy. The cell response of the biphasic CaPs was evaluated by in vitro cytotoxicity assessment using mouse fibroblast (L929) cells. The bilateral femoral defect rabbit model was used to assess the early local reaction of the coral-derived biphasic CaPs bone graft on tissue. The results confirmed that the co-existence of β-TCP and HAp was formed at 1100 °C for 1 h. The ratio of HA/β-TCP increased as the heat-treatment time increased. The coral-derived biphasic CaPs comprising 61% HAp and 39% β-TCP (defined as HT-3) were not cytotoxic. Furthermore, no significant differences in local tissue reaction were observed between the HT-3 sample and autogenous bone. Therefore, the synthesized coral-derived biphasic CaPs is a candidate for bone grafting due to its good biocompatibility.

## 1. Introduction

Coral exoskeletons possess unique interconnected porous architecture including tubular cavities ranging from 100 to 250 mm in length, similar to human bones and teeth, and have consequently attracted attention in orthopedics and maxillofacial surgery [1,2]. The presence of macropores greater than 150–500 µm in diameter facilitates nutrient diffusion, enables osteogenesis, and enhances bone formation [3]. Coral exoskeletons are crystals composed of calcium carbonate in the form of aragonite or calcite; their resorption rate as a bone growth rate is too fast to allow sufficient bone ingrowth, which limits clinical applications [4,5,6].

Hydroxyapatite (HAp, Ca_10_(PO4)_6_(OH)_2_) is more similar to bone and teeth and shows lower solubility than calcium carbonates in the body fluids [7,8,9]. Therefore, biomimetic synthesis methods have been explored to include HAp and other CaP with the calcium carbonate microstructure with the interconnected macroporosity [10,11,12,13,14]. Both in vitro and in vivo studies have demonstrated that the bioceramics from these biogenic sources have dual functions of osteoconduction and osteoinduction [13,14].

The co-existence of HAp and β-tricalcium phosphate (β-TCP, Ca_3_(PO_4_)_2_) was often observed under the routes to synthesize the pure HAp or β-TCP [15]. β-TCP has a comparable chemical composition to HAp; however, it is considered resorbable in vivo due to its faster release of Ca^2+^ and PO_4_^3−^ ions when exposed to physiological fluids [16,17]. As bone graft substitutes, mixtures of the stable HAp with Ca/P ratio of 1.67 and the soluble β-TCP with Ca/P ratio of 1.5 have demonstrated higher efficacy for degradation rate to match the new bone formation rate when compared with single-phase HAP or β-TCP [18,19,20].

Hydrothermal conversion is the preferred method for the production of coral-derived HAp. However, this synthetic process is inappropriate for commercial manufacturing because this process is slow, has complicated pH adjustments, and limited β-TCP batch size [21,22,23]. Under the solid-state reaction route, a homogeneous mixture of calcium and phosphate precursors can be mixed in water at room temperature to synthesize a biphasic CP with a controlled ratio of HAp to β-TCP using various heat-treatment temperatures and durations [21,24]. Highly crystalline and biphasic CaPs synthesized were suitable for mass production, high reproducibility, and low processing cost [25].

The present study aimed to synthesize highly crystalline biphasic CaPs from propagated *Scleractinian* coral [26] and dicalcium phosphate anhydrous (DCPA, CaHPO_4_) using a solid-state reaction followed by heat treatment. The influence was investigated of the heat-treated durations on the formation of the crystalline CaPs and their respective cytotoxicity properties. The rabbit bone defect model was used to assess the potential of coral-derived CPs as bone grafting.

## 2. Materials and Methods

### 2.1. As-Prepared Coral Granules

DCPA was used as a phosphate precursor. The propagated *Scleractinian* coral was purchased (Popeye Marine Biotechnology Limited, New Taipei City, Taiwan). Organic substances were removed using a self-developed cleaning process, and the coral was manually crushed and sieved using a 595 μm-mesh sieve. These deproteinized coral granules were washed thoroughly using the demineralized water, neutralized with phosphate-buffered saline (PBS), and sterilized in an autoclave. A commercial coral calcium powder, hereafter denoted as SMP-44 (Biomed herbal, Taichung, Taiwan), was used for comparison.

### 2.2. Coral-Derived Biphasic CaPs

The as-prepared coral granules and DCPA with the Ca to P (designated as Ca/P) molar ratio of 1.50 were mixed in demineralized water and homogenized in a brushless stirrer at a rotation speed of 450 rpm for 4 h. After filtering and drying, the mixture was heat-treated at 1100 °C in the high-temperature furnace (JH-4, Kingtech Sciencetific, Taipei, Taiwan) for a specified duration. The samples herein were referred to as HT-0.1, HT-3, and HT-7 when the heat treatment was for 1 h, 3 days, and 7 days.

### 2.3. Surface and Microstructure Analysis

Crystallinity analysis and phase identification of the heat-treated samples were performed using X-ray diffractometry (XRD; Rigaku 2200, Tokyo, Japan) operated with Cu Kαradiation operated at 50 kV and 250 mA. Crystalline phases were identified by comparing the database from the Joint Committee on Powder Diffraction Standards (JCPDS) [27]. The mass fraction was semi-quantified using the area of the peaks from β-TCP (0210) and the sum of the area of the peaks from HA (211) and β-TCP (0210). The chemical bonding information of the samples was characterized via Fourier-transform infrared spectroscopy (FTIR; Perkin-Elmer Spectrum 100, Shelton, CT, USA) with a spectral resolution of 4 cm^−1^. Surface morphology of the samples was examined using scanning electron microscopy (SEM; JEOL-6500F, Tokyo, Japan) using an accelerating voltage of 20 kV [28,29]. Raman spectra were recorded at room temperature using Raman and a scanning near-field, optical microscope equipped with a 633 nm excitation laser source (Horiba HR800, Protrustech Co., Ltd., Taipei, Taiwan) [24,30].

### 2.4. In Vitro Cytotoxicity Evaluation

The mouse fibroblast cell line (L929 RM60091, Bioresource Collection, and Research Center, Hsinchu, Taiwan) was adopted in this experiment according to ISO 10993-5 specification [31]. The cells were seeded in culture dishes at a density of 5 × 10^4^ cells per 100 μL in α-Minimum Essential Medium (MEM; Level Biotechnology, New Taipei City, Taiwan). Cells from passage 2 were harvested at 80% confluence and used for further 3-[4,5-dimethylthiazol-2-yl]-2,5-diphenyltetrazolim bromide (MTT) assay. The extracts of the investigated samples were placed in an orbital shaker maintained at 37 °C for 24 h with a mass to volume extraction ratio of 0.2 g/mL, which was followed by filtering and sealing in sterile bottles. L929 cells at a density of 1 × 10^4^ cells/well were cultured in MEM and seeded on the 24-well culture plates. After obtaining a confluent monolayer, the medium was replaced by 0.1 mL sample extracts and incubated for 24 h at 37 °C in an atmosphere of 5% CO_2_ (*n* = 3). Subsequently, a 10 μL MTT assay kit (R&D system, Minneapolis, MN, USA) was added to each well and incubated for 2 h. The optical density (OD) value of each plate was read at 570 nm using a microplate reader (ELx800, BioTek, Winooski, VT, USA). The cell viability is expressed as shown in Equation (1):

(1) $$\mathrm{Viability}\;\mathrm{rate}\;\left(\%\right)\;=\;\frac{{\mathrm{OD}}_{\mathrm{HT}}-{\mathrm{OD}}_{b}}{{\mathrm{OD}}_{\mathrm{nc}}-{\mathrm{OD}}_{b}}\times 100\%$$

where HT represents the measured OD of the samples, and nc and b represent the measured ODs of the negative control (NC) and the blank. The culture medium with 10% (*v/v*) dimethyl sulfoxide (DMSO) and an extract from high-density polyethylene were used as positive control (PC) and NC [32].

For qualitative evaluation, L929 cells at a density of 5 × 10^4^ cells/well were seeded on the 24-well culture plates and incubated for 24 h at 37 °C in an atmosphere of 5% CO_2_. The original culture medium was replaced by 0.5 mL sample extracts and incubated for 24 h at 37 °C in an atmosphere of 5% CO_2_ (*n* = 3). Then, the cells were stained with the neutral red solution (Merck Taiwan, Taipei, Taiwan). Changes in cell morphology, cell lysis, and membrane integrity were observed using the inverted fluorescence microscope (FV1000/IX81, Olympus, Tokyo, Japan) under different magnifications.

### 2.5. A Pilot Study of the Rabbit Model for In Vivo Biocompatibility Assessment

**Figure 1:**
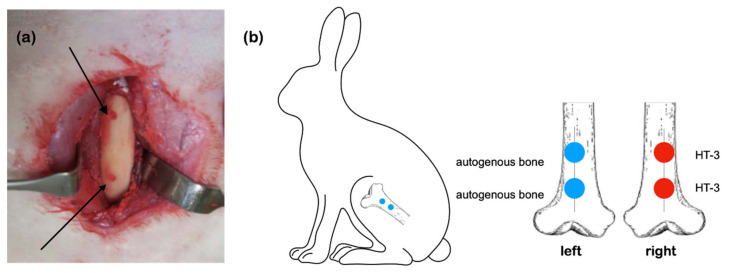
(**a**) Surgical model and (**b**) a schematic implantation design in the right distal femur of rabbit.

A pilot study was carried out at Master Laboratory Co., Ltd. (Hsinchu, Taiwan) according to the standard of ISO 10993-6:2016 “Biological evaluation of medical devices—Part 6: Tests for local effects after implantation”. Five New Zealand white rabbits weighing 2.8–3 kg (Livestock Research Institute, Tainan, Taiwan) were used to assess in vivo local tissue reaction using a bone defect model at the distal femur where autogenous bone was used for comparison. Before implantation, rabbits were sedated by intramuscular injection of tiletamine–zolazepam (10 mg/kg, Virbac Taiwan, Taipei, Taiwan) and xylazine hydrochloride (10 mg/kg, Bayer Taiwan, Taipei, Taiwan); then, they were anesthetized with isoflurane under aseptic conditions. Bone defects were created with a diameter of 4 mm and depth of 5 mm in the bilateral condyle of the femur using a motorized drill (Frios Unit S, Dentsply Sirona Taiwan, New Taipei City, Figure 1a). The bone graft sample was implanted into the defect site on the right femur, and the autogenous bone was implanted into the left femur (Figure 1b) by a well-trained veterinary. The rabbit was euthanized with carbon dioxide 4 weeks post-operatively. Bone blocks were collected from the adjacent and bottom regions of original bone defects for histological analysis. The bone blocks were fixed in 10% neutral-buffered formalin, dehydrated using ethanol, embedded in methyl methacrylate (MMA), sectioned into 4 to 5 μm-thick slices, stained with hematoxylin and eosin (H&E), and observed via using the Aperio CS pathology scanner (Leica Biosystems, Buffalo Grove, IL, USA).

### 2.6. Statistical Analysis

The SPSS statistic software (Version 19.0., SPSS Inc., Chicago, IL, USA) was used to analyze the experimental data. The difference between groups was determined by one-way analysis of variance followed by Tukey’s HSD post hoc test. A *p* value of less than 0.05 was considered as statistically significant.

## 3. Results

### 3.1. Characterizations of the Propagated Coral Granules

**Figure 2:**
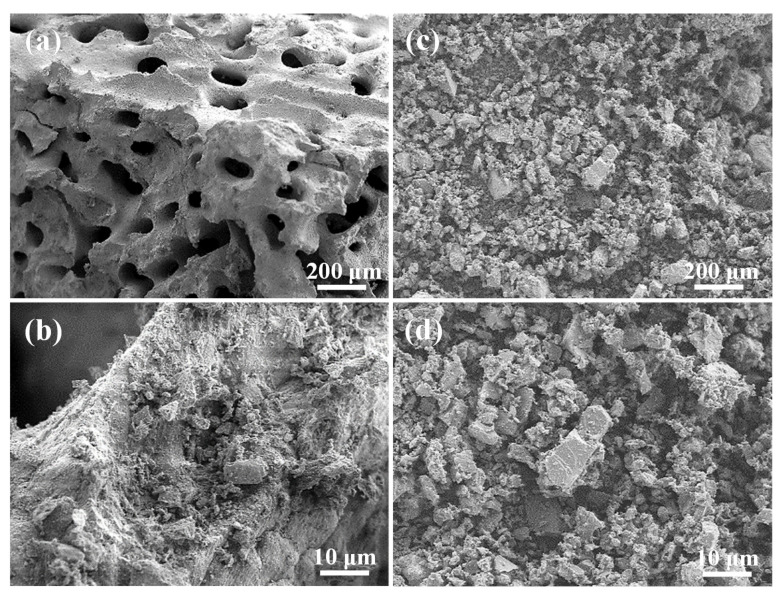
Comparisons between as-prepared coral and commercial coral calcium powder samples. (**a**) SEM image of the as-prepared coral, (**b**) a higher magnification SEM image of the as-prepared coral, (**c**) SEM image of the commercial coral calcium sample, and (**d**) a higher magnification SEM image of the commercial coral calcium sample.

The SEM image in Figure 2a reveals the as-prepared coral granules with interconnected pore sizes of 100 to 250 μm. A polycrystalline and fibrous morphology was observed at the highly magnified image; as shown in Figure 2b–d, the SMP-44 sample of commercial coral calcium powder did not have the interconnected pores.

**Figure 3:**
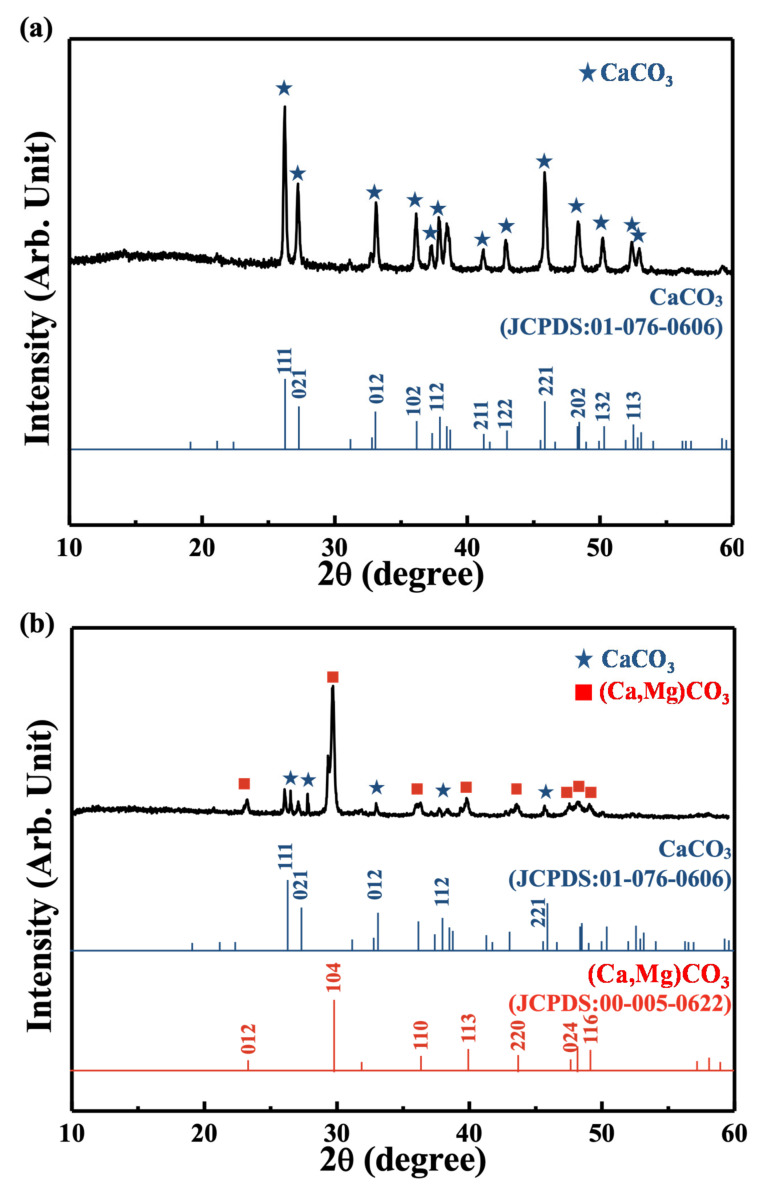
XRD pattern of (**a**) the as-prepared coral and (**b**) the commercial coral calcium sample.

Figure 3a shows that the as-prepared coral exhibited a fully crystalline, single phase of aragonite (JCPDS 01-076-0606), which is similar to the literature [4]. The diffractogram of the SMP-44 sample shown in Figure 3b showed crystalline peaks corresponding to (Ca,Mg)CO_3_, but the peaks slightly shifted to larger diffraction angles (JCPDS 00-005-0622).

**Figure 4:**
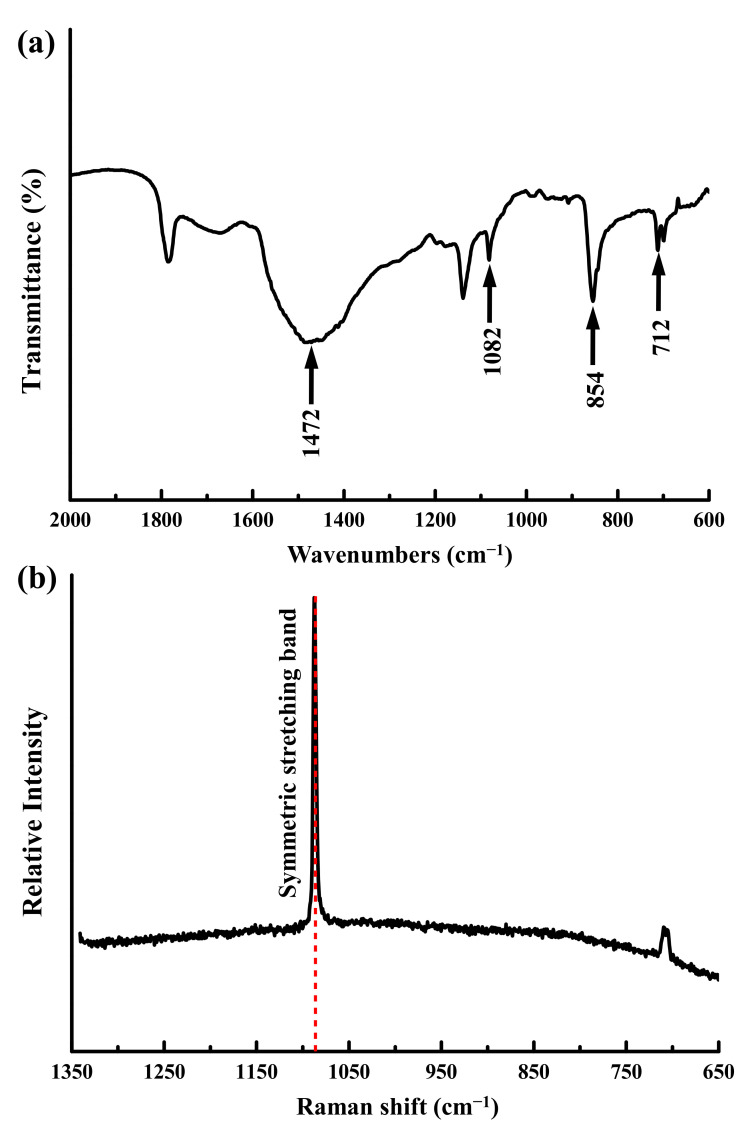
(**a**) FTIR and (**b**) Raman spectra of the as-prepared coral sample.

FTIR spectra revealed that the as-prepared coral exhibited the characteristic bands for aragonite at 712 (in-plane bending mode, ν4), 854 (out of plane bending mode, ν2), 1082 (symmetric stretching mode ν1), and 1472 (asymmetric stretching, ν3) cm^−1^, as shown in Figure 4a. The Raman spectroscopy confirmed the phases present, as shown in Figure 4b. The characteristic Raman symmetric stretching band (ν1) at 1087.6 cm^−1^ and the in-plane bending mode (ν4) at 705.8 cm^−1^ of the Raman spectra also indicate that the as-prepared coral granules were aragonite.

### 3.2. Effects of Heat-Treated Duration on the Formation of Biphasic CaPs

**Figure 5:**
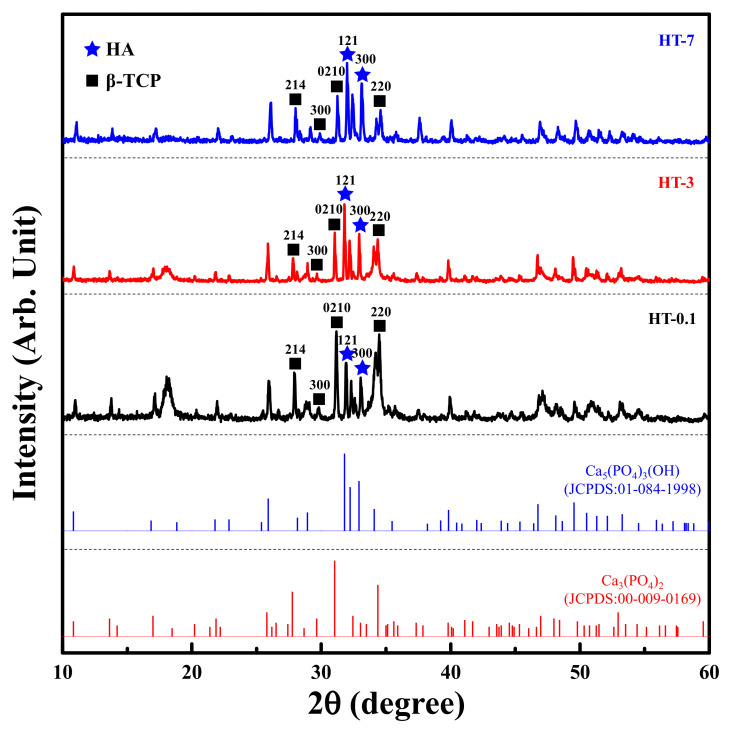
XRD patterns of the samples prepared by the heat treatment of as-prepared coral and DCPA at various durations, including reference spectra for JCPDS 01-084-1988 and 00-009-0169.

Figure 5 shows the XRD patterns of the samples prepared by the heat treatment of as-prepared coral and DCPA at various durations. The diffraction spectra of all heat-treated samples were similar. A complete decomposition of CaCO_3_ took place; these diffraction peaks were absent after heat treatment. Only β-TCP (JCPDS 00-009-0169) and HAp (JCPDS 01-084-1998) phases were observed for all heat-treated samples. The diffraction intensity of the peaks corresponding to β-TCP decreased with longer heat-treated time. Table 1 presents the results of semi-quantification of the crystalline phases in biphasic CaPs, showing that longer heat treatment created more HAp.

**Figure 6:**
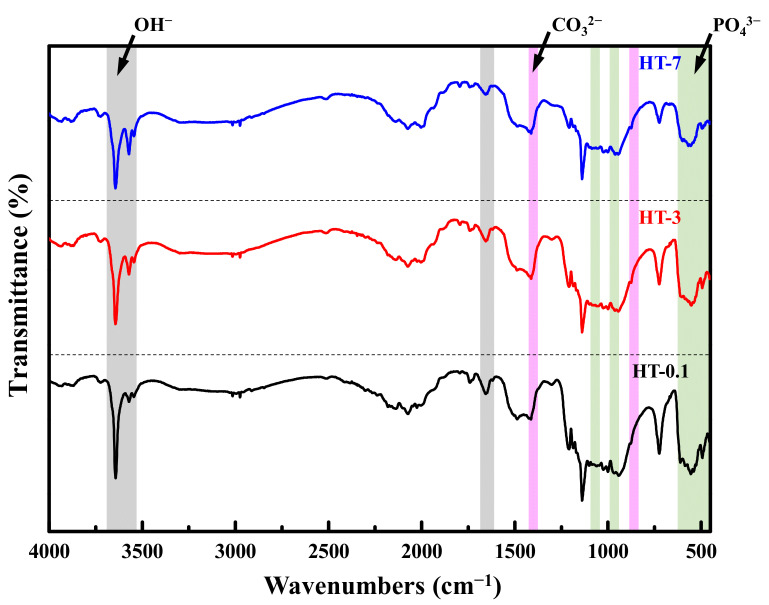
FTIR spectra of the samples prepared by the heat treatment of various durations of as-prepared coral and DCPA. Green areas indicate the bond regions of ${\mathrm{PO}}_{4}^{3-}\;$ groups; gray areas indicate the bond regions of ${\mathrm{OH}}^{-}\;$ groups; pink areas indicate the bond regions of ${\mathrm{CO}}_{3}^{2-}\;$ groups.

Figure 6 shows the effect of heat treatment duration on the FTIR spectra. The spectra of all heat-treated samples were similar, having the hydroxyl (${\mathrm{OH}}^{-}$) and phosphate (${\mathrm{PO}}_{4}^{3-}$) groups characteristic of CaPs. The sharp peak at 3643 cm^−1^, belonging to the stretching vibration motion of the ${\mathrm{OH}}^{-}$ groups, was observed for all heat-treated samples. The bond regions of ${\mathrm{PO}}_{4}^{3-}$ groups at 611–540 cm^−1^ and 1139–944 cm^−1^ were also observed in all heat-treated samples, as were the asymmetrical stretching bands ν3 (1139 cm^−1^), the symmetric stretching bands ν1 (944 cm^−1^), and the bending bands ν4 (611, 586 and 543 cm^−1^), which were related to ${\mathrm{PO}}_{4}^{3-}$ groups of β-TCP [13,29]. All samples also presented the asymmetrical P-O stretching mode (ν3) at 1086 cm^−1^, the asymmetrical bending modes (ν4) at 600 and 568 cm^−1^, and the symmetric stretching modes at 962 (ν1) and 474 (ν2) cm^−1^, which are distinguishable peaks in the ${\mathrm{PO}}_{4}^{3-}$ groups of crystalline HAp [18]. Two peaks were exhibited at 3544 cm^−1^ and 3571 cm^−1^ that represented the stretching vibrations of the ${\mathrm{OH}}^{-}$ groups in crystalline HAp. A trace of the symmetric stretching vibration of the ${\mathrm{HPO}}_{4}^{2-}$ groups at 877 cm^−1^ appeared in all heat-treated samples.

**Figure 7:**
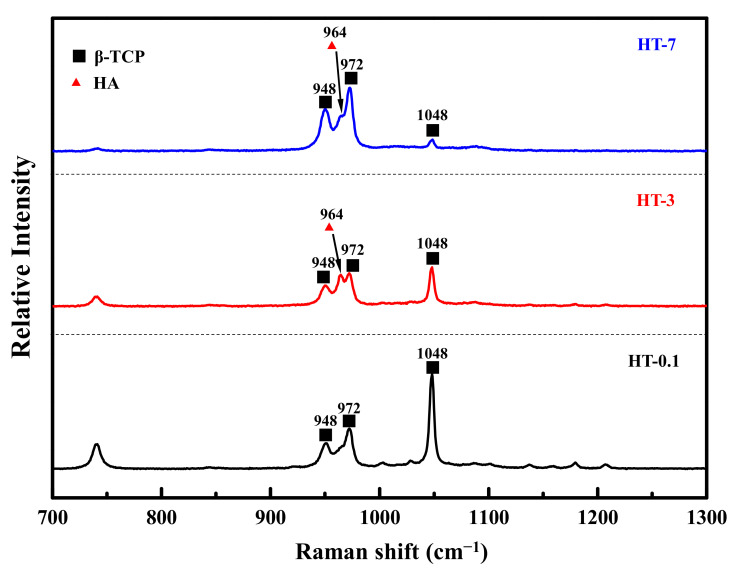
Raman spectra of the investigated samples prepared by the heat treatment of as-prepared coral and DCPA at different durations.

Figure 7 has the Raman spectra of the samples prepared by calcination of the as-prepared coral and DCPA at different durations in the fingerprint region (900–1100 cm^−1^). All heat-treated samples exhibited the main vibration modes associated with ${\mathrm{PO}}_{4}^{3-}$ groups: a wideband representing symmetric P-O stretching mode (ν1) located in 930–990 cm^−1^, and the asymmetric stretching (ν3) was at 1030–1080 cm^−1^. Some differences were observed in the range between 950 and 980 cm^−1^. The HT-0.1 sample displayed major peaks at 950, 972, and 1048 cm^−1^ that could be assigned to the vibrational internal modes of ${\mathrm{PO}}_{4}^{3-}$ groups of β-TCP. Some peaks at 964 and 1088 cm^−1^ were observed in the HT-0.1 sample, indicating the formation of HAp. With the increasing heat-treated time, the peak intensity at 964 cm^−1^ increased, and that at 1048 cm^−1^ decreased.

### 3.3. In Vitro Cytotoxicity Response

**Figure 8:**
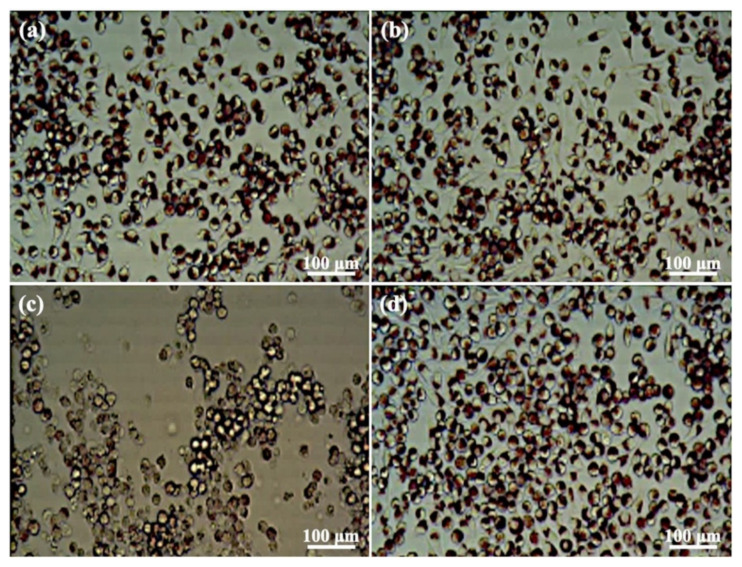
Optical images of the cell morphologies on the investigated samples: (**a**) blank, (**b**) negative control, (**c**) positive control, and (**d**) HT-3.

Based on the above investigations, the HT-3 sample was selected for the study of the heat-treatment conditions discussed in the in vitro cytotoxicity response and in vivo bone defect test. Table 2 has the OD values of cells that were co-cultured with extracts from the HT-3 sample for 24 h. No significant differences in cell viability were observed among the blank, NC, HT-3, and 50% HT-3 groups, and the cell viabilities were higher than those of the PC group. The cell viabilities of both 50% and 100% extracts of the HT-3 sample were higher than 70%, indicating that the HT-3 sample possessed no acute cytotoxic potential. Figure 8 shows the cell morphology variations of the tested samples. Cells had long spindle shapes with good density in the blank and NC groups (Figure 8a,b), whereas round cells and nearly destruction of the cell layers were observed in the PC group (Figure 8c). The HT-3 group (Figure 8d) had a cell morphology and density similar to the blank and NC groups.

### 3.4. Bone Tissue Reaction Features

**Figure 9:**
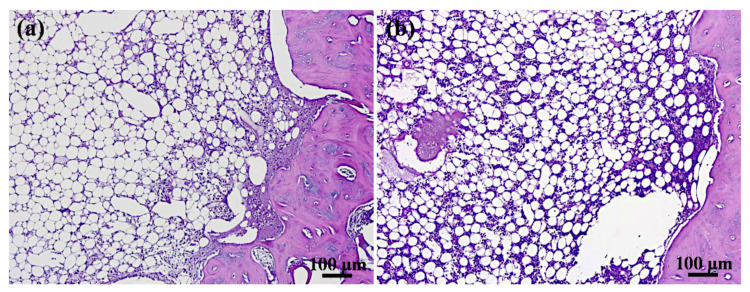
H & E staining results of the investigated samples after 4 weeks of implantation: (**a**) control and (**b**) HT-3.

Figure 9 shows the histological images of the defects. No abnormal behavior or wound infection were found in the two graft materials, the coral samples, or autogenous bone, 4 weeks after the bone graft. Lymphocytes in the HT-3 group were higher than the autogenous bone group, although no significant differences were observed in the overall tissue reaction between the two materials (Table 3). These findings indicate that the HT-3 samples were considered non-responsive to tissues compared to autogenous bone samples after 4 weeks of implantation. The investigated HT-3 sample had no adverse effect on the tissue response and was comparable to that of the autogenous bone sample.

## 4. Discussion

Coral-derived CaP composed of HAp and β-TCP were obtained after heat treating the mixture of the propagated *Scleractinian* coral and DCPA at 1100 °C for as little as 1 h. The DCPA suspension with NaOH, NaF, or NaCl was transformed into HAp or TCP after hydrolysis for seven days; however, this process was slow and had complicated pH adjustments [21,22]. Furthermore, traditional porous-forming technologies were hard and relatively rare to realize the complex structures, which were structurally similar to human bone and tooth enamel [10,11]. The present study used the propagated *Scleractinian* coral as a calcium precursor and provided the calcium carbonate microstructure with the inter-connected microporosity.

β-TCP may be formed at 800 °C through the dehydration of DCPA to β-Ca_2_P_2_O_7_ and decomposition of CaCO_3_ to CaO [21,24]. Rhee et al. [25] synthesized mixtures of HAp and β-TCP using mechanochemical treatments. No noticeable changes of the initial two powders, Ca_2_P_2_O_7_ and CaCO_3_, were observed after 8 h of milling, but the surface area increased, which caused the speculation. Similar results were observed in the present study. A dominant component of the raw material used, the propagated *Scleractinian* coral, was aragonite CaCO_3_. The heat treatment for 1 h resulted in the complete decomposition of the starting materials into only two crystalline phases: HA and β-TCP. The relative amount of β-TCP was higher than HAp after 1 h of heat treatment and decreased with longer heat treatment, implying that β-TCP was easily formed. The structural similarity between the starting materials and β-TCP facilitated the transformation from CaCO_3_ into β-TCP [16]. In additional, the Ca/P ratio of the starting materials also provided favorable conditions for β-TCP formation [17].

The relatively greater proportion of HAp after increasing heat treatments may result from the instability of β-TCP [20]. With higher heated temperature, slight hydration and a slight deficit of hydration water in DCPA facilitated to form HAp, as shown in the following reaction [25]:

$$4{\mathrm{Ca}}_{3}{\left({\mathrm{PO}}_{4}\right)}_{4}+2H_{2}O\to {\mathrm{Ca}}_{10}{\left({\mathrm{PO}}_{4}\right)}_{6}{\left(\mathrm{OH}\right)}_{2}\left(\mathrm{surface}\right)+2{\mathrm{CaHPO}}_{4}.$$

The diffraction peaks of CaP-based mixtures were overlapped because of the similar chemical structures. Therefore, the vibrational spectroscopies helped characterize vibrations for these materials’ amorphous species or crystalline phases [15,29]. The FTIR and Raman spectra results were in good agreement with the XRD findings in the present study. The presence of the Raman (Figure 5) ${\mathrm{OH}}^{-}$ peaks at 3645 cm^−1^ may result from the adsorbed moisture due to the highly hygroscopic property of phosphates and the appearance of the ambient air atmosphere during heat treatment [24]. The ${\mathrm{OH}}^{-}$ peak decreased with longer heat treatment, suggesting that the conversion of β-TCP to HA was formed due to the existence of water and the supply of additional hydroxyl groups [25].

The mixture heat-treated for 3 days (HT-3) contained 61% HAp and 39% β-TCP, and there was no significant difference in HAp/β-TCP ratio between HT-3 and HT-7 samples (Table 2). DMSO was used as a positive control because it was cytotoxic. High-density polyethylene was used as a negative control to clear the background response of the cells [32]. These results and the MTT assay demonstrated that heat treatment for 3 days was sufficient to generate biphasic CaP with the stable phase composition and excellent biocompatibility properties [16]. The results of qualitative visual cellular characterization shown in Figure 7 from the cytotoxicity test agreed with those of MTT assay analysis regarding the toxicity of the cells. The cell layer reactivity that resulted from biomaterial extracts can be scored from 0 (no reactivity) to 4 (severe reactivity) [31]. L929 cells exposed to HT-3 showed no reactivity (Grade 1) and were considered cytocompatible.

The pilot study of rabbit femur defects revealed that the HT-3 sample did not induce an irritant response 4 weeks after rabbit femur bone implantation. It had the same biological effect (without adverse effect) as the autogenous bone sample. Therefore, it is believed that the coral-derived biphasic CaPs comprising 61% HAp and 39% β-TCP (i.e., HT-3 sample) not only exhibited good biocompatibility but also possessed the potential to facilitate bone tissue regeneration. More studies should be performed to validate the present findings.

## 5. Conclusions

Biphasic calcium phosphates containing β-TCP and HAp were synthesized by stirring solutions of propagated coral and dicalcium phosphate anhydrous followed by heat treatments at 1100 °C for 1 h to 7 days. The synthesized biphasic CaPs samples displayed no cytotoxic effects. Implantation in bone defects in the rabbit model did not induce locally adverse tissue reactions and had new bone formation. The coral-derived biphasic CaPs with 61% HAp and 39% β-TCP sample are a useful bone graft substitute for bone defect treatment applications.

## Figures and Tables

**Table 1:** Relative phase content (%) in biphasic CaP synthesized for varying durations.

Phase	HT-0.1	HT-3	HT-7
HAp	39.8	61.0	62.6
β-TCP	60.2	39.0	37.4

**Table 2:** The results of MTT assay for evaluation of cell viability.

	OD_570 nm_	Viability (%)	Cell Lysis (%)
Blank	0.986 ± 0.002	100	0
NC	0.984 ± 0.003	100	0
PC	0.098 ± 0.001	10	90
HT-3	0.929 ± 0.035	94	6
50% HT-3	0.963 ± 0.028	98	2

**Table 3:** Histological findings and irritant-ranking scores according to ISO 10993-6: 2016.

	HT-3 (*n* = 10)	Autogenous Bone Group (*n* = 10)	*p* Value
Polymorphonuclea	1.2 ± 1.8	0.8 ± 1.1	0.68
Lymphocytes	4.4 ± 0.9	3.6 ± 0.9	0.20
Plasma cells	0	0	
Macrophages	0	0	
Giant cells	0	0	
Necrosis	0	0	
Neovascularization	0	0	
Fibrosis	0.6 ± 0.5	0.6 ± 0.5	>0.05
Fatty infiltrate	2.2 ± 0.5	2.4 ± 0.9	0.67

## Data Availability

Data are contained within the article.
